# Controlled Release of Diclofenac from Matrix Polymer of Chitosan and Oxidized Konjac Glucomannan

**DOI:** 10.3390/md9091649

**Published:** 2011-09-23

**Authors:** Suphat Korkiatithaweechai, Pornpusadee Umsarika, Narong Praphairaksit, Nongnuj Muangsin

**Affiliations:** Department of Chemistry, Faculty of Science, Chulalongkorn University, 254 Phayathai Road, Bangkok 10330, Thailand; E-Mails: suphat.top@gmail.com (S.K.); jp_pu_24@hotmail.com (P.U.); narong.pr@chula.ac.th (N.P.)

**Keywords:** drug delivery system, diclofenac sodium, chitosan, oxidized konjac glucomannan

## Abstract

The controlled release of diclofenac sodium (DFNa) from a chitosan-oxidized konjac glucomannan (CTS-OKG) polymer film was studied. Konjac glucomannan (KGM) was initially oxidized by sodium periodate and then cross-linked to CTS via imine bonds (–C=N–) to form the new CTS-OKG copolymer. The DFNa loaded CTS-OKG polymers were characterized by Fourier transformed infrared spectroscopy (FT-IR) and X-ray diffractometry (XRD). Finally, the release profiles of DFNa from the CTS-OKG polymer matrices were evaluated in a simulated gastrointestinal fluid system comprised of two hours in simulated gastric fluid (SGF; pH 1.2) followed by 24 h in simulated intestinal fluid (SIF; pH 7.4). A 1:2:1 (w/w/w) ratio of CTS:OKG:DFNa prepared at room temperature for 3 hours gave the highest % encapsulation efficiency (EE) of 95.6 ± 0.6 and resulted in a minimal release of DFNa (<1% over 2 h) in SGF (pH 1.2) and a significantly improved sustained release in SIF (pH 7.4) with ~6% and 19% release over 8 and 24 h, respectively), some 15- and five-fold lower than that of the two commercial DFNa preparations, Diclosian and Voltaren. This formulation may be used for further study as a long term intestine controlled release drug model (at least 3 days).

## 1. Introduction

Chitosan (CTS) is the deacetylated form of chitin, a natural polymer from the exoskeleton of crustaceans, insects and cell walls of fungi and is as a waste product from marine aquaculture products like prawns/shrimps. CTS has received considerable attention as a functional, sustainably renewable, nontoxic and biodegradable biopolymer with low immunogenicity and bactericidal activity for diverse applications, especially in pharmaceutics, food and cosmetics [1]. Drug controlled release is a scheme to increase the efficiency of treatment as the concentration of active substance is regulated continuously and/or at more appropriate sites. Natural polymers are the products of living organisms and are often readily available, sustainably renewable, and possess better biocompatibility, biodegradability, a low or non-toxicity, and have a higher modification capability compared to various synthetic materials. Diclofenac sodium (DFNa) is a widely used non-steroidal anti-inflammatory drug (NSAID) for the treatment of rheumatoid arthritis, osteoarthritis and ankylosing spondylitis. Besides high costs, the most common side effects of DFNa are undesirable, being gastric ulcers, gastrointestinal bleeding, blood dyscrasia, anaphylaxis and depression of renal function, making more efficient delivery systems that avoid the initial high dose spikes highly desirable [2,3]. In this scenario, the ideal system will protect the drug with minimal release in the acidic gastric environment, but with a complete off-loading of the DFNa at a sustained slow release in the intestine.

**Scheme 1:**
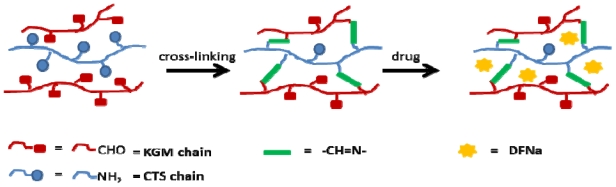
Formation of a chitosan-oxidized konjac glucomannan (CTS-OKG) polymer film via the Schiff-base reaction and hydrogen bond.

KGM is high-molecular weight, water-soluble, non-ionic and natural polysaccharide [4]. Chemically, it is a linear random copolymer of β-(1,4), linked d-mannose and d-glucose at a molar ratio of 1.6:1. Being a β-(1,4) linked polysaccharide, KGM can be oxidized by reacting with sodium periodate to yield OKG. Simultaneously, the carbon–carbon bonds of the *cis*-diol group in the KGM molecular chain are cleaved and generate reactive aldehyde functions, which can chemically cross-link with CTS via imino bonds, or Schiff base’s linkage, between the free amino groups of CTS and the aldehyde groups of the OKG (shown in Scheme 1). The OKG has been developed as a potentially less toxic cross-linking agent for the gelatin-based biometerials [5] and the chitosan-based hydrogels [6]. The novel hydrogels prepared from oxidized konjac glucomannan cross-linked chitosan for *in vitro* drug delivery using ofloxacin as a model drug. The hydrogels show good swelling properties in both pH 2.2 and 7.4 and give the sustained release profiles with about 84% released of ofloxacin in within 7 h at pH 2.2 medium [6].

In this paper, we report on the oxidation of konjac glucomannan (KGM) with periodate to form the oxidized product (OKG) as a macromolecular cross-linking agent for CTS to replace the traditional toxic glutaraldehyde and prepare composite polymer films for controlled release drug delivery.

## 2. Results and Discussion

### 2.1. Synthesis and Characterization of Oxidized Konjac Glucomannan (OKG)

**Scheme 2:**
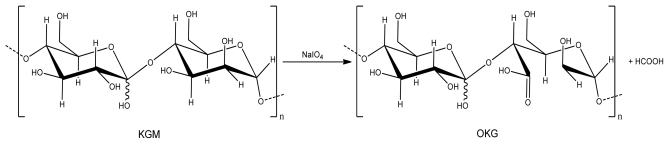
Oxidation reaction of konjac glucomannan (KGM) with NaIO_4_ to yield Oxidized Konjac Glucomannan (OKG).

KGM is a linear random copolymer of β-(1,4) linked d-mannose and d-glucose. The vicinal hydroxyl groups of KGM can be cleaved by periodate oxidation to form dialdehyde derivatives (Schemes 1 and 2).

**Figure 1:**
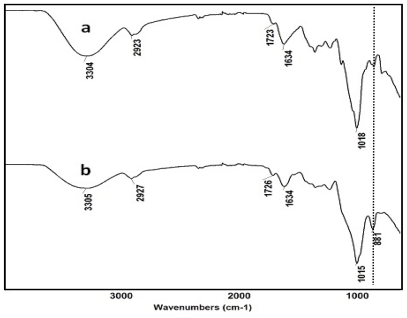
Representative infrared spectroscopy (FT-IR) spectra of (**a**) the KGM used in this study and (**b**) its oxidized derivative, OKG.

According to the literature [7], linear polysaccharides easily undergo the non-specific oxidation based cleavage of the glycosidic bonds of the backbone polymer. The oxidation reaction of KGM by sodium periodate can be monitored with attenuated total reflectance Fourier transformed infrared spectroscopy (FT-IR). Figure 1 shows the representative FT-IR spectra of the native KGM used in this study and its derived OKG.

In the spectrum of the native KGM, the adsorption band at 1723 cm^−1^ is ascribed to the carbonyl moiety of the acetyl group whilst the intense peak at 1634 cm^−1^ is attributed to the in-plane deformation of the water molecule [8]. However, the oxidation of KGM to OKG leads to the appearance of two characteristic bands of OKG at around 1726 cm^−1^ and 881 cm^−1^. The former is also ascribed to the aldehyde symmetric vibrational band (carbonyl), which changed from the small shoulder seen in the native KGM to this distinct peak in OKG. The latter peak (881 cm^−1^) is assigned to the hemiacetal structure between the aldehyde group and neighboring hydroxyl groups [9].

#### 2.1.1. Characterization of the CTS-OKG Polymer Film

**Figure 2:**
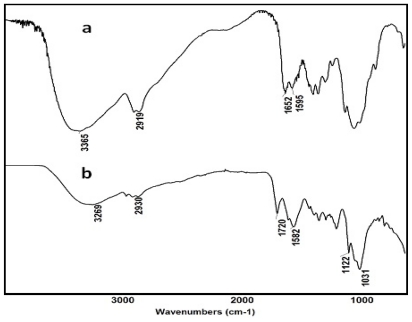
Representative FT-IR spectra of (**a**) CTS and (**b**) a 1:1 (w/w) ratio CTS:OKG polymer film.

Once the OKG was successfully prepared, the composite CTS-OKG polymer (1:1 (w/w) ratio CTS:OKG) films were prepared by a Schiff’s base cross-linking reaction between the amine groups of CTS and the aldehyde groups of OKG. Representative FT-IR spectra of CTS and a CTS-OKG polymer from a 1:1 (w/w) ratio of CTS:OKG are shown in Figure 2.

In the case of the native CTS, the wide absorption band at 3365 cm^−1^ is ascribed to the stretching vibration of O–H bonded to N–H, whilst the bands appearing at 1652 cm^−1^ and 1595 cm^−1^ are ascribed to NH_2_ bending vibration mode. From the FT-IR spectra of the 1:1 (w/w) ratio CTS:OKG composite CTS-OKG polymer film, a new peak at about 1720 cm^−1^, ascribed to the C=N stretching vibration of the imine group of a Schiff base, is observed, whilst the CTS peaks at 1652 cm^−1^ and 1595 cm^−1^ and the characteristic OKG hemiacetal structure peak at 881 cm^−1^ disappear. This confirms the formation of coupling reaction between the –CHO groups of OKG and the NH_2_ of CTS.

**Figure 3:**
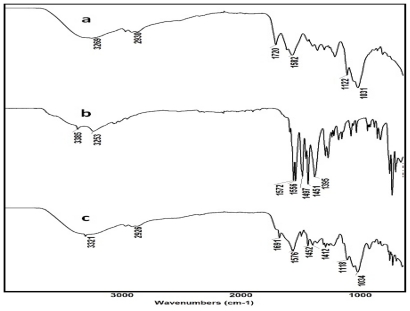
Representative FT-IR spectra of (**a**) the CTS-OKG polymer (1:2 (w/w) ratio CTS:OKG), (**b**) DFNa and (**c**) the 3OKG Diclofenac sodium (DFNa)-loaded CTS-OKG polymer (1:2:1 (w/w/w) ratio CTS:OKG:DFNa).

In order to confirm the possibility of a structural change in the encapsulated DFNa in the DFNa-loaded CTS-OKG polymer film, its FT-IR spectra was compared to that for the CTS-OKG polymer without DFNa and with DFNa alone. The FT-IR spectra for the 3OKG polymer film (1:2:1 (w/w/w) ratio CTS:OKG:DFNa) is shown in Figure 3 as the representative DFNa-loaded CTS-OKG polymer, but the spectra for the other six DFNa-loaded CTS-OKG polymers of different compositions (see Table 1) show the same characteristic peaks (data not shown).

The FT-IR spectra of the CTS-OKG polymer with a 1:2 (w/w) ratio CTS:OKG seen in Figure 3 was essentially identical to that of the CTS-OKG polymer with a 1:1 (w/w) ratio CTS:OKG (Figure 2), as expected. For DFNa, the wide absorption bands around 3385 cm^−1^ to 3254 cm^−1^ were due to the stretching vibration of the N–H group. The band appearing at 1574 cm^−1^ is due to the carbonyl (C=O stretching) of the carboxylate group. In case of the FT-IR spectra of the DFNa-loaded CTS-OKG composite polymer (3OKG), the peaks of DFNa are present at 3321 cm^−1^, 1576 cm^−1^, 1452 cm^−1^ and 1412 cm^−1^ in addition to all other peaks normally present in the CTS-OKG composite polymer. Therefore, the above results indicated that the CTS-OKG composite polymer and DFNa did not react with each other.

#### 2.1.2. X-ray Diffraction (XRD)

**Figure 4:**
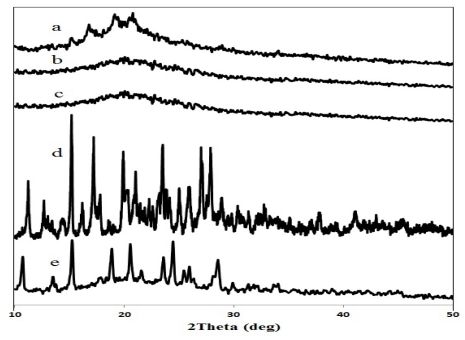
Representative X-ray diffractograms of (**a**) CTS, (**b**) OKG, (**c**) CTS-OKG composite polymer (1:1 (w/w) ratio CTS:OKG), (**d**) DFNa and (**e**) the DFNa-loaded CTS-OKG polymer (1:2:1 (w/w/w) ratio CTS:OKG:DFNa).

In order to investigate the crystallinity of the composite polymers and the stability of DFNa after loading into the CTS-OKG composite polymers, X-ray diffractograms of the DFNa-loaded CTS-OKG composite polymer (1:2:1 (w/w/w) ratio CTS:OKG:DFNa) were compared with those of the CTS, OKG, CTS-OKG composite polymer (1:1 (w/w) ratio CTS:OKG) and DFNa (Figure 4).

As observed, the XRD pattern of CTS presents three major crystalline peaks at a 2θ of 16.72, 19.04 and 20.4°. The OKG exhibited an amorphous structure as no diffraction peak was observed. However, the crystalline peaks of CTS entirely disappeared, and an amorphous state was observed in the CTS-OKG composite polymer, suggesting that the formation of the Schiff’s cross-linking structure between the amino groups of CTS and the aldehyde groups of OKG suppresses the crystallization of CTS. Given that the crystal structure of CTS is stabilized by hydrogen bonds between the amino groups and hydroxyl groups [5], then the strong cross-linking action between CTS and OKG molecules likely reduces the internal hydrogen bonding interactions in CTS leading to the appearance of an amorphous structure in the composite polymers.

Many sharp peaks were observed in the XRD patterns of DFNa, at a 2θ of 11.22, 15.18, 19.90, 23.46 and 24.42°, which indicate that DFNa is crystalline. When comparing the XRD patterns of the CTS-OKG polymer matrix with that for the DFNa-loaded CTS-OKG matrix, the characteristic peaks of DFNa were change to diclofenac acid in the form of *C2/C* [10].

### 2.2. Encapsulation of DFNa in the CTS-OKG Polymer Films

The effects of the varying the CTS:OKG:DFNa weight ratios on the DFNa entrapment efficiency were investigated and the results are shown in Table 1 in terms of the % encapsulation efficiency (EE) of DFNa.

From Table 1, formulation 3OKG has the largest % EE of about 95.6%, whilst formulation 2DFNa has the lowest % EE of about 39.4%.

With respect to the weight ratio of CTS to OKG, when the OKG content was increased from 1:0 to 1:2 (CTS:OKG) at a fixed DFNa ratio, the % EE increased in a dose-dependent manner from about 53% to 95%. Thus, although OKG crosslinking of CTS is not required for the encapsulation of DFNa, its presence increases the % EE (and the sustained release rate (data nor shown), and this was probably because the more OKG that was used, the more crosslinking occurred, leading to higher amounts of DFNa being entrapped.

With respect to the proportion (as a (w/w) ratio) of CTS in the formulation, as the CTS content was increased from 0.5:1 to 1:1 (CTS:OKG) with a fixed DFNa ratio, the % EE was increased significantly from about 44% to 73%, but increasing the CTS content further to a 2:1 (w/w) CTS:OKG caused a slight decrease in the % EE from about 73% to 68%. This is consistent with the above results that when the proportion of CTS is more than that for the OKG, the amount of crosslinked sites is lower and hence a lower amount of DFNa is entrapped yielding a lower % EE.

With respect to the weight ratio of DFNa to CTS-OKG, when the DFNa content was increased from 0.5:1 to 1:1 (w/w) DFNa: CTS-OKG, the % EE was increased from about 39% to 73%, but further increasing the DFNa content from 1:1 to 2:1 (w/w) decreased the % EE from about 73% to 39%. This is probably because the excess DFNa relative the amount of CTS-OKG could not be entrapped in the CTS-OKG polymer matrix.

### 2.3. *In Vitro* Release Study

#### 2.3.1. The Effect of the OKG Weight Ratio

**Figure 5:**
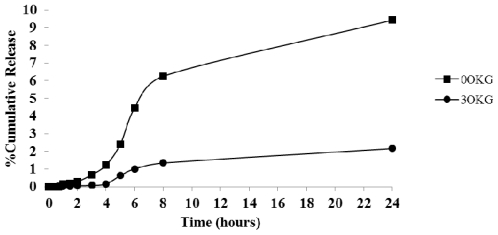
Time-dependent DFNa released from DFNa-loaded CTS and CTS-OKG polymer film in SGF (pH 1.2). Data are shown as the mean ± SD and are derived from three repetitions.

In order to investigate the pH sensitive of the CTS-OKG polymers, the release profile of diclofenac in SGF (pH 1.2) from diclofenac-loaded CTS and CTS-OKG films at 37 ± 2 °C for 24 h were performed. The release profiles are shown in Figure 5.

**Figure 6:**
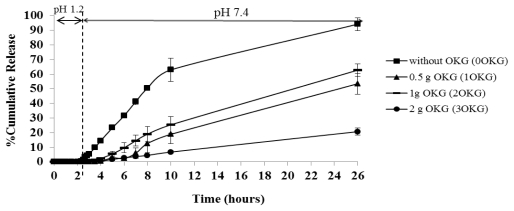
Time-dependent DFNa release from DFNa-loaded CTS-OKG polymer film formulations with varying CTS-OKG (w/w) ratios in simulated gastric fluid (SGF) (pH 1.2) and simulated intestinal fluid (SIF) (pH 7.4). Data are shown as the mean ± SD and are derived from three repetitions.

The cumulative release profiles of DFNa from the CTS and CTS-OKG polymer film revealed that both polymer films can prolong release of diclofenac. After two hours, the release rate of diclofenac from the CTS film is faster than that from the CTS-OKG and significantly different after the sixth hour (about 1% for CTS-OKG and 6% for CTS). The results suggested that the CTS-OKG can more retard the release of diclofenac in SGF than that of CTS film even, the solubility of diclofenac sodium is very low in the acidic condition. This is attributed from the amino groups in the CTS polymer film have been protonated to –NH_3_^+^ in the SGF (pH 1.2), resulted in the repulsions between the amino groups and allowed the water molecule to penetrated in the polymer matrix, hence swollen as shown in Figure 6.

**Scheme 3:**
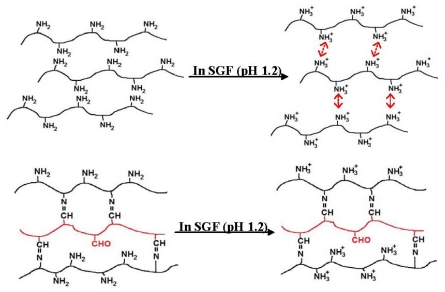
CTS polymer film and CTS-OKG polymer film in SGF (pH 1.2).

In case of the CTS-OKG polymer film, some of amino groups of CTS reacted with aldehyde groups of OKG to give imine bonds, so the amine groups in CTS-OKG polymer film are less than that in the native CTS, consequently, less swollen. Therefore CTS-OKG polymer film can more control the release of DFNa from the polymer film in SGF (pH 1.2) (Shown in Scheme 3).

The release profiles of DFNa from the DFNa-loaded CTS-OKG polymers formulated with varying amounts of OKG were evaluated in a crude simulated gastrointestinal system of using a 2 h exposure to simulated gastric fluid (SGF; pH 1.2) followed by 24 h in simulated intestinal fluid (SIF; pH 7.4), all at 37 °C. The results are shown in Figure 6.

The cumulative release profiles of DFNa from encapsulation in the different CTS:OKG (w/w) ratio polymer films revealed a sustained DFNa release profile in all cases. In common, all formulations released almost no DFNa in the SGF (pH 1.2) at less than 1% in 2 h, but the release rates of DFNa from the formulations in SIF (pH 7.4) varied with their CTS:OKG proportions. The formulation without OKG (0OKG) showed by far the fastest DFNa release rate of all the samples, with DFNa release starting within the first hour of exposure to SIF (pH 7.4) and attaining a rate of around 6.2% of the total DFNa level released per hour over the first 8–10 h. A relatively much slower release of DFNa was recorded from the OKG containing composites, with negligible DFNa release in the first two hours at pH 7.4. Thereafter, the order of the DFNa release rate was (highest to lowest) OKG > 1OKG >> 3OKG polymers, with an average DFNa release rate over the 2–8 h period in pH 7.4 of about 3.2%, 2.1% and 0.75% of the total DFNA released per hour, respectively. In the subsequent period of 8–24 h at pH 7.4 (10–26 h total assay time) DFNa was still released from all composites but at a slower rate, and attained approximately 90% total release rate in the OKG free formulation (0OKG) compared to ~60%, 50% and 19% for the 2OKG, 1OKG and 3OKG composites, respectively.

The high OKG proportion (2:1 (w/w) OKG:CTS in 3OKG) in the polymer matrix causes a delay in the kinetics of DFNa release, presumably due to the increased crosslink density that was present. However, that the formulation 2OKG that contains a two-fold higher proportion of OKG than formulation 1OKG gave a slightly higher DFNa release rate might be due to the higher amount of CTS (1:0.5 (w/w) CTS:OKG). In this scenario, the molar excess of then free CTS amine groups remaining in the matrix can form strong hydrogen bonds in the polymeric chains, helping to delay the release rate of the DFNa. These results are consistent with the results obtained when varying the proportion of CTS (section 2.3.2). When the proportion of OKG was higher than that for CTS (1:2 (w/w) ratio of CTS:OKG), the DFNa release profiles may thus be dependent upon the hydrogen bonding between the hydroxyl groups of OKG and the amine groups of CTS.

#### 2.3.2. The Effect of the CTS Weight Ratio

**Figure 7:**
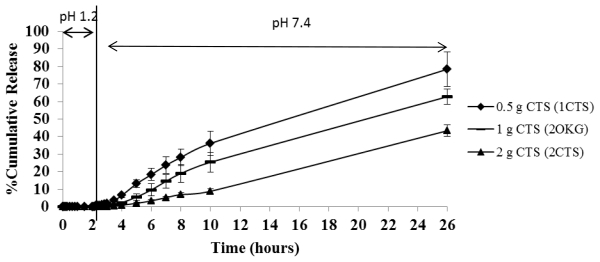
Time-dependent DFNa release from DFNa-loaded CTS-OKG polymer formulations with varying amounts of CTS:OKG but a fixed 1:1 OKG:DFNa ratio, in SGF (pH 1.2) and SIF (pH 7.4). Data are shown as the mean ± SD and are derived from three repetitions.

As mentioned in section 2.3.1, the proportion of OKG in the formulation clearly affected the DFNa release profile. Therefore, the relative proportion of CTS is likely to be an important factor and was investigated. The release profiles of DFNa from the DFNa-loaded CTS-OKG polymer formulations with varying amounts of CTS (from 0.5:1 to 2:1 (w/w) CTS:OKG) at a fixed 1:1 (w/w) ratio of OKG:DFNa were evaluated in the same simulated gastrointestinal system (SGF (pH 1.2) for 2 h and then SIF (pH 7.4) for the next 24 h). The results are shown in Figure 7.

As observed before (section 2.3.1), DFNa was hardly released at all from all three DFNa-loaded CTS-OKG polymer composites in the SGF buffer (less than 1% of the total amount of loaded DFNa over the 2 h). In the SIF buffer, the DFNa release rates for the three formulations were either very small (<4%; 1CTS) or negligible (2OKG and 2CTS) in the first two hours, and then showed a relatively slow release of DFNa from the CTS-OKG polymer matrix, in the order of 1CTS > 2OKG >> 2CTS (containing 0.5:1, 1:1 and 2:1 (w/w) ratios of CTS:OKG, respectively) over the next six hours (2–8 h total time in pH 7.4) averaging 7.0%, 4.3% and 1.8%, or 5.8%, 3.8% and 1.3% of the total DFNa released per hour over these 4 and 6 h, respectively. Thereafter, DFNa was still released from all three formulations, with around 78%, 60% and 41% of the total DFNa loaded being released by 24 h in pH 7.4.

The results indicated that the DFNa release rate is affected by the CTS content in the CTS-OKG polymer matrix. A high CTS content causes a delay in the kinetics of DFNa release, because, as the proportion of CTS is increased so is the crosslink density and then also so is the amount of free amine groups for hydrogen bonding with the OKG hydroxyl groups increased.

#### 2.3.3. The Effect of the DFNa Weight Ratio

**Figure 8:**
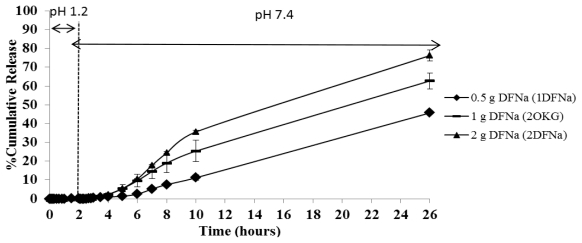
Time-dependent DFNa release from CTS-OKG polymers (1:1 (w/w) ratio CTS:OKG formulation) with varying amounts of DFNa when immersed in SGF (pH 1.2) followed by SIF (pH 7.4). Data are shown as the mean ± SD and are derived from three repetitions.

In the view of the fact that the amount of DFNa in the CTS-OKG polymer may affect its release rate, the release profiles of DFNa from DFNa-loaded CTS-OKG polymer formulations with varying amounts of DFNa from 0.5:1 to 2: 1 (w/w) DFNa:CTS-OKG were evaluated with a 1:1 (w/w) fixed weight ratio of CTS:OKG, using the same simulated gastrointestinal system at 37 °C. The results are shown as the DFNa cumulative release profiles in Figure 8.

As observed before (sections 2.3.1 and 2.3.2), almost no DFNa was released from the three DFNa-loaded CTS-OKG polymers in the two hours in SGF (<1% total level of loaded DFNa) and in the first two hours in the SIF. Thereafter, DFNa was released at a slow rate from all three CTS-OKG polymer compositions over the next six hours (4–10 h total time), with the rate of DFNa release being related to the amount of DFNa loaded in the composite, in the order (highest to lowest release rate) of 1DFNa > 2OKG > 2DFNa with average release rates over the 6 h from 4 to 10 h total time of approximately 5.7%, 4.0% and 1.7% of the total DFNa loaded per hour for 1DFNa, 2OKG and 2DFNa, respectively, and a total release after 26 h of ~73%, 61% and 43%, respectively.

Thus, OKG as a crosslinking polymer with CTS can control the DFNa release at pH 1.2 to a minimal level over 2 h but maintain a sustained release of DFNa over more than 8 h in pH 7.4 media, releasing from at least 43 to 73% of the loaded dose of DFNa.

**Figure 9:**
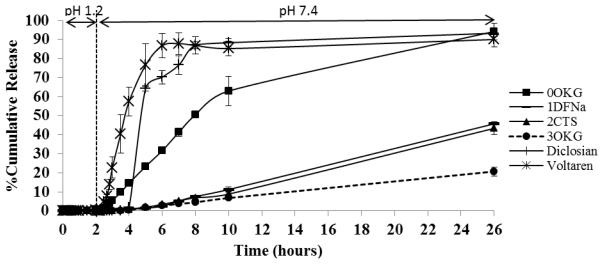
Time-dependent DFNa release from the various vectors in SGF (pH 1.2) and SIF (pH 7.4) at 37 °C. Data are shown as the mean ± SD and are derived from three repetitions.

Finally, the formulations for each effect that gave the minimum and maximum DFNa release in the SGF (pH 1.2) and SIF (7.4) buffers, respectively, were selected to compare with that for the two commercial DFN drugs (Diclosian and Voltaren tables) in the same mock gastrointestinal system (Figure 9).

In common with the DFNa-loaded CTS-OKG polymers noted before (sections 2.3.1–2.3.3 and Figure 9), both commercial drugs (Diclosian and Voltaren) released less than 1.0% of the total DFNa loaded in the SGF dissolution medium over 2 h. However, in contrast to the DFNa-loaded CTS-OKG polymers when immersed in the SIF dissolution medium, the commercial drugs released DNFa faster and sooner with a significant release amount of ~35%, ~65% and >87% of the total amount of DFNa loaded being released from both formulations after one, two and four hours in pH 7.4, respectively. As presented before (section 2.3.1), the CTS matrix without OKG (0OKG) presented a significantly faster DFNa release than the OKG containing CTS-OKG polymers, but this was still slower than that of the two commercial tablets taking more than 10 h to release >90% of the DFNa. Comparing the DFNa release from the four different DFNa-loaded CTS-OKG polymers, it is clear that the release rate is affected by the CTS, OKG and DFNa proportions in the polymer matrix.

In addition, the release of DFNa from the DFNa-loaded CTS-OKG composite polymers is also clearly depended on the pH of the release medium, with essentially no release in the SGF buffer (pH 1.2) over two hours compared to the significant release in the SIF buffer (pH 7.4). Release rates in the SGF were not assayed beyond two hours as the stomach contents are retained in the stomach for about 2 h before passing to the duodenum.

The DFNa release rates are summarized for all composites in Table 2. In comparison with the % EE of DFNa (Table 1), it can be deduced that the formulation 3OKG had the slowest release rate and gave the highest % EE. Therefore, the 3OKG formulation may be appropriate for further study as a model drug controlled release model for a longer period of time, such as three days released model, although it remains to be established that the total DFNa released would improve from the 19% seen at 24 h to a more acceptable 50% at 3 days. Alternatively, for shorter sustained delivery models, for example, formulation 2OKG would still offer benefits over the two commercial DFNa drugs.

## 3. Experimental

### 3.1. Material

The following materials were obtained from the indicated suppliers and used as received. DFNa was from the Center for Chitin-Chitosan Biomaterial, Thailand. CTS (food grade, deacetylation 90% min., M.W. 50–300 kDa) was obtained from BFM, Bangkok, Thailand. Hydrochloric acid 35%, sodium hydroxide, sodium hydrogen phosphate, potassium chloride, potassium dihydrogen phosphate, potassium periodate and potassium bromide were all from Merck, Germany. Lactic acid and KGM (food grade, % glucomannan >95%, M.W. above 1 × 10^6^) were from Union Chemical, Bangkok, Thailand. Diclofenac and Voltaren commercial DFNa tablets were obtained from Asia pharmaceutical, Bangkok, Thailand and Novartis Co., Ltd. (Thailand).

### 3.2. Synthesis of Oxidized Konjac Glucomannan (OKG)

KGM was oxidized using sodium periodate to OKG as follows. Into 500 mL of 1% (w/v) KGM was introduced 5 g of sodium periodate, and the mixture was stirred vigorously in the dark for 24 h at room temperature. Ethylene glycol (50 mL) was then added to the reaction mixture to reduce any unreacted periodate and stirred for another 2 h. The reacted mixture was then dialyzed against 250 mL of distilled water (dialysis tube, MW cutoff 12 kDa) with several changes of the water over 3 days until the dialysate was free from iodate, as checked with silver nitrate. The oxidized reaction product (OKG) was recovered from the supernatant by precipitation with excess acetone and harvested by filtration three times with resuspension in 50 mL of each time. The samples were dried and stored in vacuum desiccators over silica gel until further use.

### 3.3. Preparation of the DFNa-Loaded CTS-OKG Polymer Films

To a solution of 1 g of OKG in 10 mL distilled water was added the required quantity of CTS, dissolved in 1% (v/v) lactic acid, to obtain the desired weight ratio of CTS:OKG. Then, the desired amount of DFNa was added into the polymer mixture and stirred vigorously. Finally, the solution mixture of CTS/OKG/DFNa was poured into a plastic tray and air dried. The films were peeled off and randomly punched by paper punch with the punch size of 3/8 inch or 11.1 cm, after that the punched films was pulverized and sieved through the sieve with openings of 1 mm.

The weight ratios of the seven different formulations of the DFNa-loaded CTS-OKG polymer composites used in this study are shown in Table 1.

### 3.4. Characterization of the CTS-OKG Polymer Films

#### 3.4.1. FT-IR Analysis

To confirm the formation of the cross-linked structure and find the chemical stability of the encapsulated DFNa in the CTS-OKG polymer matrix, the attenuated total reflectance Fourier transform infrared spectra (FT-IR) of all samples was measured (Nicolet 6700). Various samples were scanned in range between 4000 and 400 cm^−1^. Spectra were signal averaged over 32 scans with a resolution of 4 cm^−1^ at room temperature.

#### 3.4.2. X-ray diffraction (XRD)

Wide-angle XRD patterns of each sample were analyzed using a XRD Rigaku DMAX 2200 Ultima+ diffractrometer, equipped with CuKα target at 40 kV and 30 mA, at a scan rate of 5°/min. The diffraction angle ranged from 2θ = 10° to 50°.

### 3.5. *In Vitro* DFNa Release Study

The DFNa release from the polymer films was evaluated in a shaking incubator at 50 rpm and 37 °C. The desired quantity of DFNa-loaded CTS-OKG polymer films (0.1 g) was immersed in 250 mL of release medium comprised of either simulated gastric fluid (SGF, pH 1.2) or simulated intestine fluid (SIF, pH 7.4) as the dissolution media. At scheduled time intervals, 5 mL of solution was withdrawn and an equal volume of the same dissolution medium was added back to maintain a constant volume. The amount of DFNa released from the CTS-OKG polymers was determined by UV-Visible spectrophotometric measurements at 276 nm (Perkin Elmer, Lambda 800, USA) and calculated by reference to the DFNa ncalibration standard curves within the concentration range of 1–10 ppm of *y* = 0.028*x* + 0.0088 (*R*^2^ = 0.9999) for pH 1.2 and *y* = 0.0317*x* + 0.0139 *(R*^2^ = 0.9998) for pH 7.4. The results are the means of three determinations.

## 4. Conclusions

The controlled release of DFNa from CTS-OKG polymers was evaluated in SGF (pH 1.2) for two hours followed by SIF (pH 7.4) for 24 h. The DFNa release properties could be modulated and correlated by varying the proportions of CTS, OKG and DFNa. The 1:2:1 (w/w) ratio of CTS:OKG:DFNa (preparation 3OKG) prepared at room temperature for three hours showed the slowest DFNa release rate and gave the highest % EE, and so may be appropriate to use for further study as a long-term intestine controlled release drug (DFNa) model.

## Figures and Tables

**Table 1:** Compositions of DFNa-loaded CTS-OKG polymer films and the % EE of DFNa in the CTS-OKG polymer films.

Samples	CTS (g)	OKG (g)	DFNa (g)	%Encapsulation 1
**0OKG**	1	–	1	53.05 ± 0.54
**1OKG**	1	0.5	1	54.61 ± 0.76
**2OKG**	1	1	1	73.18 ± 0.22
**3OKG**	1	2	1	95.62 ± 0.58
**1CTS**	0.5	1	1	44.54 ± 0.22
**2CTS**	2	1	1	68.12 ± 0.25
**1DFNa**	1	1	0.5	39.77 ± 0.16
**2DFNa**	1	1	2	39.37 ± 0.05

1 Data are shown as the mean ± SD and are derived from three repetitions.

**Table 2:** Summary of the DFNa release kinetics from the different DNFa-loaded CTS-OKG polymers plus the two commercial DFNa preparations. (Neg = neglect).

Samples	CTS:OKG:DFNa (w/w/w)	Cumulative DFNa release (% total)				
		SGF	SIF			
		2 h	2 h	6 h	8 h	24 h
0OKG	1:0:1	neg	14.51	50.37	62.95	94.22
1OKG	1:0.5:1	neg	neg	12.62	18.86	53.48
2OKG	1:1:1	neg	neg	18.97	25.37	62.77
3OKG	1:2:1	neg	neg	4.47	6.73	20.69
1CTS	0.5:1:1	neg	6.40	28.13	36.13	78.44
2CTS	2:2:1	neg	neg	6.93	8.83	43.34
1DFNa	1:1:0.5	neg	neg	7.58	11.21	45.75
2DFNa	1:1:2	neg	neg	24.39	35.62	76.26
Diclosian	--	neg	64.32	87.82	88.30	93.28
Voltaren	--	neg	57.38	86.89	85.24	89.99
